# Supplementary material for: Sorafenib-Induced Apoptosis in Hepatocellular Carcinoma Is Reversed by SIRT1
Source: Int J Mol Sci. 2019 Aug 19;20(16):4048. doi: 10.3390/ijms20164048 (PMC6719220; doi:10.3390/ijms20164048)
Supplement: Supplementary file 1 [file ijms-20-04048-s001.pdf]

Supplement

Sorafenib-induced apoptosis in hepatocellular carcinoma is reversed by SIRT1

A) B)

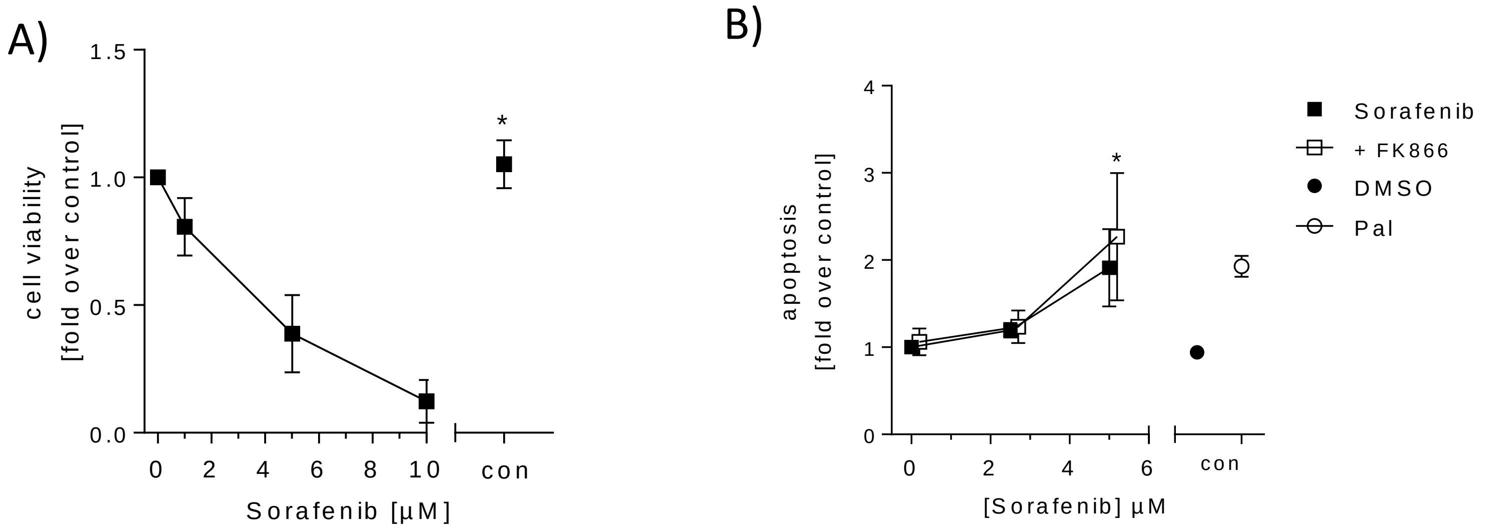

S1 Sorafenib induces apoptosis in HepG2 cells which is not further enhanced by FK866 A) WST-1 assay was performed to measure changes in cell viability in HepG2 cells after treatment with sorafenib (1µM; 5 µM and 10 µM) (n=3). B) Cells were stimulated with sorafenib (2.5µM and 5µM) alone or in combination with FK866 (10 nM) for 24 h. Apoptosis was determined by FITC Annexin V Apoptosis Detection Kit and flow cytometry. Annexin V-FITC positive cells and Annexin-V-FITC and PI positive cells were defined as apoptotic cells (n=3). The mean value of serum-free medium (0 µM) was used to normalize values of the respective datasets. Data are shown as mean ± SEM. con: solvent control (DMSO); *p<0.05.

A)

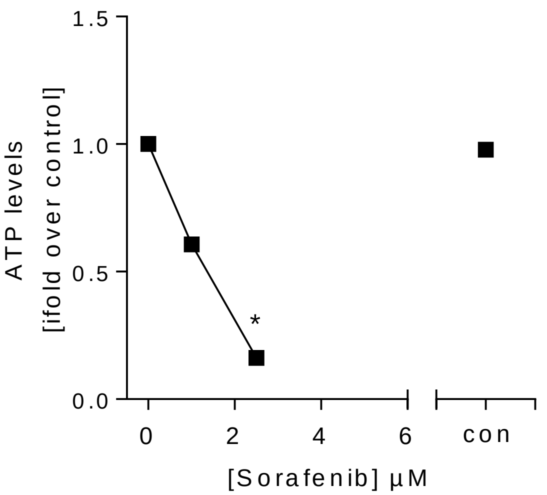

B)

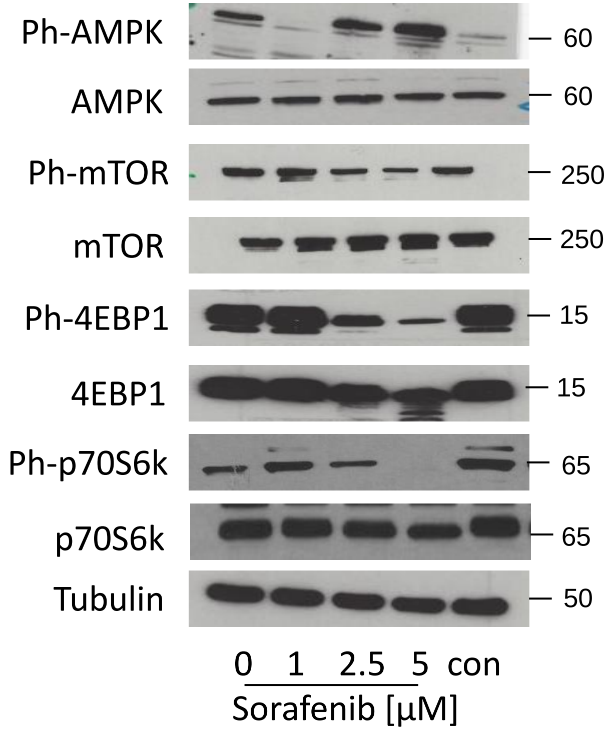

S2 Sorafenib decreases ATP concentrations and regulates phosphorylation of the AMPK/mTOR pathway in Hep3B cells.

Hep3B cells cells were stimulated with sorafenib (1 µM; 2.5µM and 5 µM) for 24h. A) ATP was determined by CellTiter-Glo Luminescent Cell Viability Assay (Promega) B) The mean value of serum-free medium (0 µM) was used to normalize values of the respective datasets. One representative blot out of 3 experiments of phosphorylated and total AMPK, mTOR, 4E-BP1 and p70S6K is shown. Tubulin served as loading control.

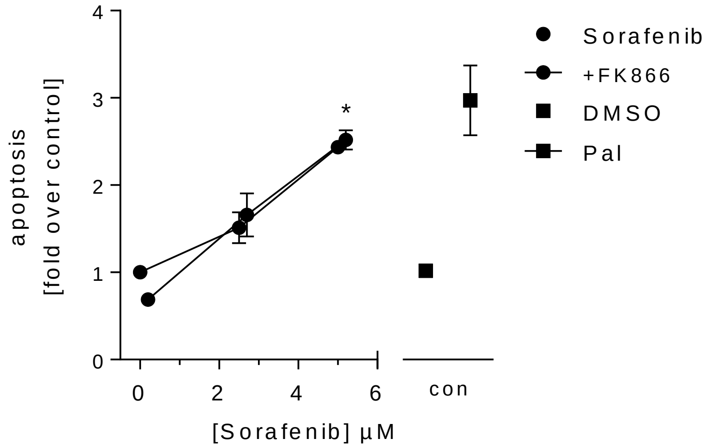

S3 FK866 does not sensitize Hep3B to sorafenib treatment.

A) Cells were stimulated with sorafenib (2.5µM and 5µM) alone or in combination with FK866 [10 nM] for 24 h. Apoptosis was determined by FITC Annexin V Apoptosis Detection Kit and flow cytometry. Annexin V-FITC positive cells and Annexin-V-FITC and PI positive cells were defined as apoptotic cells (n=3). The mean value of serum-free medium (0 µM) was used to normalize values of the respective datasets. Data are shown as mean ± SEM. con: solvent control (DMSO); *p<0.05.

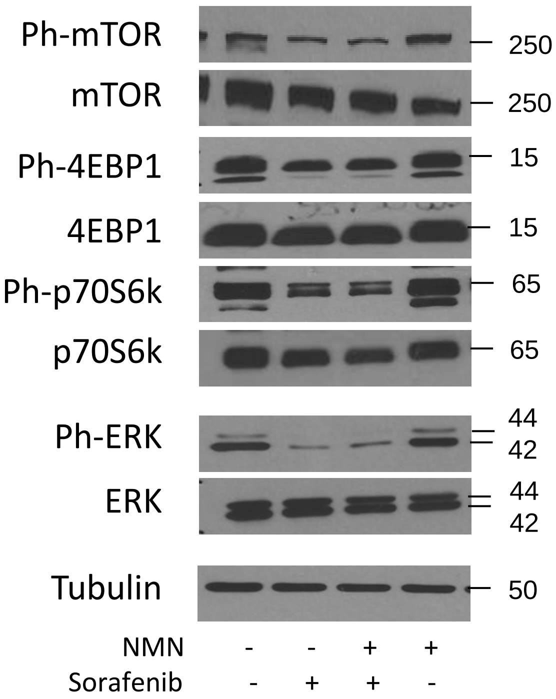

S4 NMN supplementation could not reverse effects of sorafenib on the mTOR pathway or ERK. A) HUH7 cells were stimulated with sorafenib (5 µM), NMN (250 µM) or a combination of both. Western blot analysis of phosphorylated and total mTOR , 4EBP1, p70S6K and ERK1/2 was performed. Tubulin served as loading controls, respectively. One representative blot out of 3 experiments of is shown.

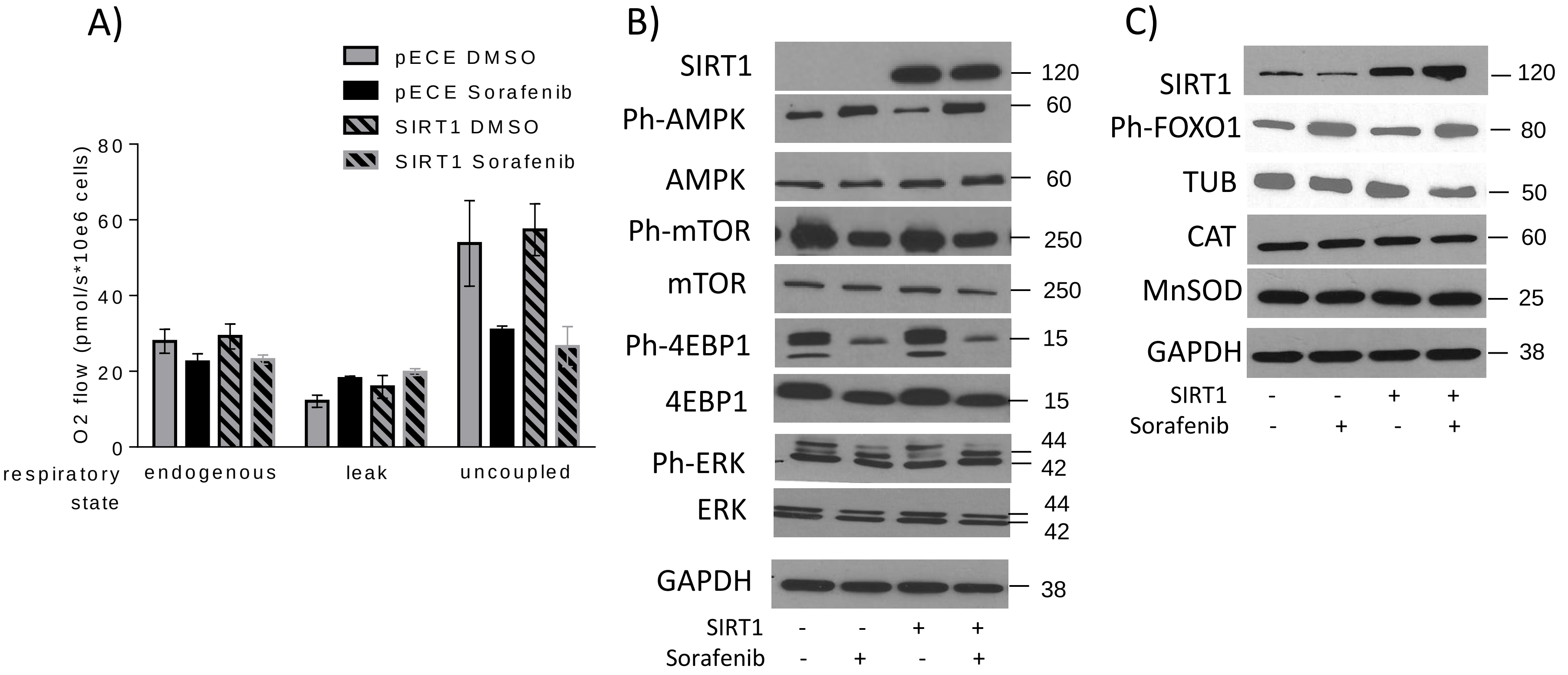

S5 SIRT1 overexpression could not reverse effects of sorafenib on mitochondrial respiration or analysed protein levels A) Mitochondrial respiration was measured by high resolution respirometry (Oxygraph2K, Oroboros) in intact HUH7 cells overexpressing SIRT1 or in vector controls (pECE) after incubation with sorafenib (1 µM for 24h) or DMSO control. Respiration was measured endogenously and after sequential inhibition of ATP synthase with Oligomycin (Leak) and uncoupling by FCCP (ETS). Residual O_2 consumption after adding Complex III inhibitor Antimycin A was subtracted. Data are presented as mean ± SEM. n = 2 for DMSO, n = 3 for Sorafenib

B) SIRT1 overexpressing HUH7 or vector control cells were incubated with sorafenib (5 µM for 24h) or DMSO control. Western blot analysis of SIRT1, phosphorylated and total AMPK, mTOR , 4EBP1, and ERK1/2 was performed. GAPDH served as loading control. One representative blot out of 3 experiments of is shown.

C) SIRT1 overexpressing HUH7 or vector control cells were incubated with sorafenib (5 µM for 24h) or DMSO control. Western blot analysis of SIRT1, phosphorylated FOXO (ph-FOXO), CATALASE (CAT), MnSOD and loading controls (TUBULIN, TUB; GAPDH) was performed. One representative blot out of 3 experiments of is shown.
